# The Global Impact of Asthma in Adult Populations

**DOI:** 10.5334/aogh.2412

**Published:** 2019-01-22

**Authors:** Oladunni Enilari, Sumita Sinha

**Affiliations:** 1 Wake Forest Baptist Medical Center, Winston-Salem, NC, US; 2 Albert Einstein College of Medicine, Bronx, NY, US

## Abstract

Asthma is a disease characterized by chronic airway inflammation, leading to intermittent symptoms of wheeze, dyspnea, cough and chest tightness in combination with variable expiratory airway obstruction. Clinical diagnosis is usually established based on the presence of symptoms and documented variability in expiratory airflow limitation as measured by pulmonary function testing. Presently, asthma is a major chronic disease affecting approximately 334 million people worldwide. The epidemic spares no age group, race or ethnicity; however ethnicity and socioeconomic status do influence the prevalence, morbidity and mortality of asthma in the United States and various countries throughout the world. Moreover, asthma places a huge burden at the societal, financial and health-care levels of multiple nations.

## Introduction

Asthma is a disease characterized by chronic airway inflammation, leading to intermittent symptoms of wheeze, dyspnea, cough and chest tightness in combination with variable expiratory airway obstruction [1]. Clinical diagnosis is usually established based on the presence of symptoms and documented variability in expiratory airflow limitation as measured by pulmonary function testing [2]. Presently, asthma is a major chronic disease affecting approximately 334 million people worldwide [3]. The epidemic spares no age group, race or ethnicity; however ethnicity and socioeconomic status do influence the prevalence, morbidity and mortality of asthma in the United States and various countries throughout the world [4]. Moreover, asthma places a huge burden at the societal, financial and health-care levels of multiple nations [2,5,6,7].

Asthma is more common in children and the leading cause of chronic airway disease; however, new onset disease can occur at any age [2,8,9]. With increasing age, it becomes more difficult to differentiate adult onset asthma from other diagnoses such as chronic obstructive pulmonary disease (COPD) or Asthma-COPD overlap syndrome (ACOS), leading to frequent under or misdiagnosis [2,7,10]. However, distinguishing between asthma, COPD and ACOS is important, not only to ensure appropriate treatment, but also for risk stratification as patients with ACOS have more exacerbations and worse prognosis [2].

## Global Epidemiology of Asthma

Accurately estimating the incidence and prevalence of asthma on a global scale is challenging because the diagnosis is often based on survey responses to questions about relatively non-specific symptoms which are open to subjective interpretation [11,12,13]. A recent multicenter cohort study conducted in Canada that enrolled 701 randomly selected adults with physician-diagnosed asthma, showed that current asthma could not be confirmed in 33% of patients [14]. Moreover, there is no universally accepted definition nor is there a single test to definitively diagnose asthma [4]. Additionally, asthma has been increasingly recognized as a heterogeneous disease comprised of both allergic and non-allergic phenotypes, a feature not captured in prior surveys.

Despite these limitations, validated tools for asthma diagnosis are available. The International Study of Asthma and Allergy in Childhood validated questionnaire, which was used in 56 countries among children aged 6 to 14 years, is one of the frequently used tools for identifying asthma in children [13,15]. Similarly, a validated instrument for adults is based on the European Community Respiratory Health Survey questionnaire [11,13]. In the United States, the National Health Interview Survey (NHIS) conducted by the Centers for Disease Control and Prevention (CDC) routinely collects data about prevalence using self-reported symptoms (such as history of wheezing) using validated measures [16].

Prior studies have shown that the prevalence of asthma ranges from 15% to 20% in many countries, especially in the developed nations [12,13]. In the United States (US), the current prevalence of asthma among adults is approximately 7.6%, but rates vary dramatically among different ethnic groups. Prevalence is 9.1% among Black non-Hispanics and 13.6% among Puerto Ricans but only approximately 5% for Mexican and Asians [16,17]. Internationally, most data on ethnic-related differences in asthma prevalence come from the United Kingdom (UK) or Canada. Netuveli et al. reported similar rates of asthma, but increased risk for admissions, among black and South Asian populations in the UK [18,19]. Wang et al. found that Chinese children born in Canada had higher rates of asthma than those who were born in China [20].

**Figure 1:**
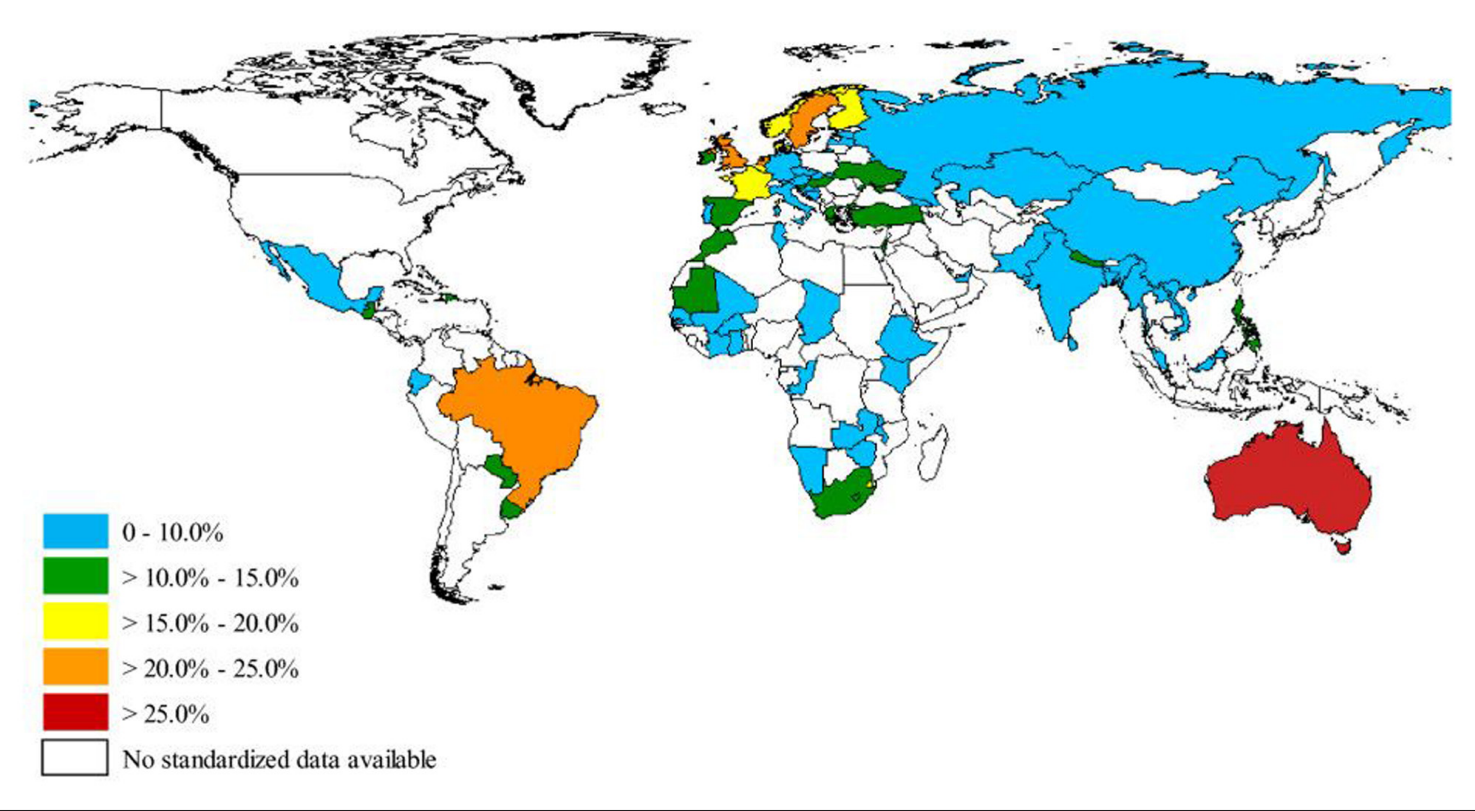
Prevalence of symptoms of asthma in the past 12 months among persons aged 18 to 45 years in 70 countries, Worlds Health Survey 2002–2003. Source: To T, et al. BMC Public Health 2012.

The World Health Survey (WHS), a standardized questionnaire designed by the World Health Organization (WHO), collected data on the prevalence of several medical conditions in adults aged 18–45 years from multiple countries around the world (Figure 1). Countries with the highest prevalence of clinical asthma were Australia (21.5%), Sweden (20.2%), UK (18.2%), Netherlands (15.3%) and Brazil (13.0%); however, the US and Canada were excluded [21]. The lowest rates were observed in Vietnam (1.0%), Bosnia-Herzegovina (1.4%), and China (1.4%) [21]. The higher prevalence observed in more developed countries may be due to increased urbanization/westernized lifestyle, higher rates of obesity, and/or pollution.

The lower asthma prevalence rates in Asia and Africa, are thought to be more related to environmental and lifestyle causes than genetic differences [15]. Individuals who reside in developed countries have a higher asthma prevalence compared to their counterparts with similar genetic predispositions but who live in developing countries or immigrated at an older age [15]. Other hypotheses that could explain lower prevalence in developing countries like China, include the lower rates of atopy, more breastfeeding, larger household size and sometimes, rural residency during childhood [6]. The “hygiene hypothesis” which has been implicated in lowering the risk of asthma, suggests that exposure to microbes and higher rates of cross infection early in life can activate type 1 helper cells, thus offsetting inflammatory pathways associated with asthma development. Conversely, improved hygiene leads to higher rates of asthma by reducing early exposure to infections [6,22]. As documented by WHO’s Study on Ageing and Adult Health (SAGE), there is substantial under-diagnosis of asthma in some less developed countries like India and Mexico, a factor that may also potentially explain lower prevalence rates compared to more developed nations [23].

Trends suggest increasing asthma prevalence globally, with an anticipated 100 million new cases in the next decade [5,24,25,26]. According to the National Center for Health Statistics, the total prevalence of asthma in US adults increased from 7.3% to 8.4% from 2001 to 2010; similar patterns have been observed among children [27,28,29]. Multiracial individuals, women, children and people of low socioeconomic status are the groups at higher risk of asthma [13,27]. Notably, there was a wide racial disparity in incidence during 2001 to 2007, with Non-Hispanic black and Mexican-American children having significantly increased rates of asthma [29]. Fortunately, more recent data suggest possible changes in these trends with decreasing childhood asthma rates and a reduction in black-white racial disparities in asthma morbidity in the US [29].

Patterns of asthma prevalence in European countries have been mixed. In Italy, De Marco, et al. reported a 35% increase in asthma prevalence in adults aged 20–44 years [30]. A parental survey for Greek schoolchildren showed an increase in asthma from 1991 to 2003 with a subsequent plateau in 2003 to 2008 [31]. Similar patterns have been reported in the Netherlands and Norway [32,33]. Conversely, a study of the UK National Health database found that the incidence of asthma was decreasing, especially in children, while lifetime asthma rates for adults continued to increase [34]. Several studies have reported increasing rates of asthma in less developed countries in Asia and Africa. Factors such as smoking, urbanization and increasing population, ingestion of seafood, and presence of moist air with subsequent mold and mildew formation, have been proposed as mechanisms explaining these trends [35,36].

## Morbidity and Mortality

**Figure 2:**
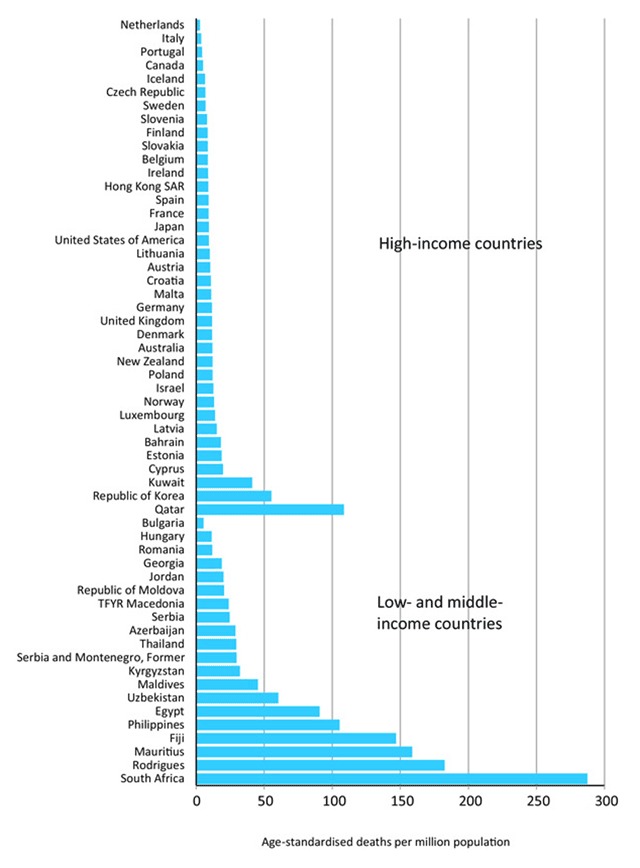
Age-standardised asthma mortality rates for all ages 2001–2010 from countries where asthma is separately coaded as a cause of death, ordered by mortality rate and country income group.* Source: WHO Detailed Mortality Database, February 2014 update.

Mortality from asthma is low compared to other chronic diseases and accounts for less than 1% of deaths globally. However, given the high prevalence worldwide, asthma is still responsible for 250,000 potentially preventable deaths annually [3,8,13,26]. In the US, mortality is highest among African Americans and Puerto Ricans and individuals of Cuban descent [37]. According to the WHO mortality database, South Africa had the highest age-standardized asthma mortality among the low and middle-income countries, while Netherlands had the lowest among the high-income countries [3,35] (Figure 2).

Though the mortality is low, asthma still leads to frequent acute healthcare resource utilization. Asthma is the 28th cause of loss of years in full health [11]. In the US alone, asthma exacerbations account for approximately two million emergency room visits each year [37]. Disease control plays a role in frequent emergency room visits as patients with severe persistent disease, while representing only 5% of the asthma population, consume over half of asthma-related resources [38,39]. Ironically, these patients only represent 5% of the asthma population [39]. Similar findings of increased healthcare utilization have been shown in Asia; in a survey-based study on over 3,000 adults and children, more than 40% of participants had at least one hospitalization or emergency room visit for asthma exacerbation. Both hospitalizations and emergency room visits were correlated with increasing level of severity [15]. Similarly, a Spanish study found that the 4% of patients with severe persistent asthma had considerable higher healthcare utilization than patients with a moderate disease [38]. On a global scale, better disease control would likely reduce associated economic costs since emergency room visits and hospitalizations account for most of the asthma-related expenses [15].

Asthma also accounts for the loss of over 15 million disability adjusted life years (DALY) annually and ranks among the highest causes of DALY for children [6,7,8]. Children aged 10–14 years, followed by elderly patients have the highest DALYs [3]. Conversely, adults aged 30–34 years have the least disability from the disease.

Morbidity due to asthma is underappreciated in the elderly. Asthma affects 7–9% of the US population older than 65 years, and causes substantial morbidity, as older adults are four times more likely to die from asthma [40,41,42]. Moreover, elderly patients have twice the risk of hospitalization and experience longer asthma-related hospital stays [43,44]. There is limited data on the prevalence of asthma in the elderly population worldwide due to lack of consensus on diagnostic criteria and diagnostic challenges due to comorbidities [3,10]. Thus, additional population-based studies are needed to better characterize the extent of this problem globally.

## Cost of Asthma Care

Among chronic diseases, asthma is one of the main contributors to increased health care expenditures (Table 1) [39,45]. The costs of asthma care can be direct monetary or indirect [45,46]. Office visits, hospitalizations, emergency room visits, cost of tests and medications all contribute to direct costs [45]. In the US, an estimated $1,500 is spent per emergency room visit [47], and over $3,000 is spent per year per patient on medical expenses alone [48]. This is in contrast to PLN$251 (approximately US$63) spent on medications in Poland in 2012 [49,50]. Conversely, Canadian patients with mild to moderate asthma are estimated to spend CAD$134 per three months (approximately US$103) on asthma care, mostly on outpatient treatment [50,51]. Indirect costs include missed work days, waiting times and productivity loss [45,46,52,53]. Ojeda et al. estimated that adults had 1.5 missed days per month due to asthma symptoms and 4.9 days per month of reduced productivity [52]. Uncontrolled asthma causes more lost productivity compared to patients with well-controlled symptoms, with a loss of approximately CAD$286 (US$219) per week [50,54]. In Spain, approximately 285 Euros (US$315) were attributed to lost workdays per patient each month [50,52].

**Table 1:** Annual Direct Costs of Asthma Care in US$ and by Severity of Illness.

Category	Mild disease (n = 140)		Moderate disease (n = 116)	Severe disease (n = 77)	Total (n = 333)

Drugs	253 ± 276	*	473 ± 310	559 ± 340	400 ± 329
General Practitioner Visits	18 ± 23		26 ± 27	39 ± 34	26 ± 29
Specialist Visits	60 ± 56		81 ± 142	82 ± 119	72 ± 107
Hospitalization	119 ± 501	*	366 ± 927	480 ± 1247	289 ± 884
Emergency Room Visits	35 ± 75		58 ± 95	75 ± 119	52 ± 95
Diagnostic Tests	48 ± 74		46 ± 74	42 ± 77	46 ± 76
Total	533 ± 833	*	1050 ± 1323	1277 ± 1703	885 ± 813

Data are mean +/– SD. *p < 0.05 for differences according to severity of disease. Adapted from Serra-Battles J, et al. Eur Resp J. 1998; 12: 1322–1326.

Many countries do not collect data on costs of asthma, especially low- and middle-income nations whose focus is primarily on infectious disease surveillance [55]. In 2009, a systematic review on the economic burden of asthma assessed 68 studies including data from the US, Canada, Europe, and Asia. Hospitalization and medication costs accounted for most of direct costs while work and school loss comprised the majority of the indirect costs [45]. However, the most recent Global Strategy for Asthma Management and Prevention (GINA) report describe that on a global scale, medication cost is the main contributor to asthma expenditures [56].

Some patient-related factors that have been implicated in higher asthma care costs include level of literacy, knowledge, beliefs, increased disease severity, poor asthma control, presence of multiple comorbidities and female sex [45]. Institutional factors that can contribute to higher asthma costs include admission to teaching or referral hospitals and intensive care unit use [45]. These findings can be due to the fact that these institutions provide care for more ill, complex patients which could lead to longer hospital days and higher need for expensive medications resulting in higher charges [45].

## Strategies to Reduce Asthma Burden

Strategies have been implemented globally to reduce asthma burden [3]. In 2013, approximately 25% of countries worldwide reported having some form of national plan for adults and/or children suffering from asthma [3]. Implementation of a national plan seems to be beneficial as demonstrated by Finland, a country estimated to have saved 300-600 million Euros a year after instituting a national initiative for asthma control [3]. This 10-year program involved a multidisciplinary approach including physicians, nurses and pharmacists [57]. It focused on educating primary care providers on asthma being an inflammatory disease, thus requiring early appropriate treatment [57]. The adoption led to practice changes among healthcare providers including routine use of peak flow monitoring, early identification and treatment and pharmacist’s education on proper inhalation technique [57]. These measures led to a decreased emergency room visits by 24%, a 54% reduction in hospital days and 76% drop in asthma-related disability [57]. Other successful results have been documented in Poland, Brazil, and Portugal [3].

In an effort to improve lung health, the Global Alliance against Chronic Respiratory Diseases (GARD) has set up guidelines to address asthma and other chronic respiratory diseases which include: determining disease burden, increasing awareness, formulating simple, affordable, and feasible policies, as well as identifying evidence-based strategies that can be easily disseminated [25]. The strategies developed by the Global Initiative for Asthma also include updating the diagnosis and treatment of asthma and modifying guideline recommendations to the local needs [2]. It is important to focus on attainable goals, frequently reviewing and modifying them to fit the problems for the particular country of interest [2,3]. Limited knowledge by both providers and patients, clinician’s busy schedules and unwillingness to adopt new measures, inadequate resources, and patient’s cultural beliefs are a few potential barriers that can prevent implementation of these strategies [2,3]. Creating work groups that includes various stake holders, including patients and providers, may help address knowledge gaps [2].

## Conclusions

Asthma is still a highly prevalent chronic disease that contributes to preventable healthcare resource utilization throughout the world. Prevalence remains high especially in developed countries and may be increasing in some low and middle-income countries. However, as many countries do not report prevalence or do not have accurate statistics, the true global burden of asthma is difficult to determine. Thankfully, mortality due to asthma is low; however, morbidity is a major problem. The most severe asthma patients account for the greatest expenditures including hospitalizations and emergency room visits. Global health organizations have identified strategies to lower asthma burden, but barriers to implementation, such as providers’ insufficient knowledge of recommendations, inertia, and lack of time and resources, remain a challenge.
